# The role of somatic NLRP3 mosaicism and new gene discovery in mutation negative cryopyrin-associated periodic syndrome patients

**DOI:** 10.1186/1546-0096-12-S1-P70

**Published:** 2014-09-17

**Authors:** Sonia Melo Gomes, Joan Arostegui, Ebun Omonyinmi, Eva Gonzalez-Roca, Ariane Standing, Dorota Rowczenio, Sira Nathanpisal, Claire Murphy, Despina Elephteriou, Nigel Klein, Philip Hawkins, Helen Lachmann, Paul Brogan

**Affiliations:** 1 Pediatric Rheumatology, Institute of child health, LONDON, UK; 2 Immunology, IDIBAPS, Barcelona, Spain; 3 Immunology, Nac RFH, Institute of child health, LONDON, UK; 4 Infection Inflammation and Immunology, Institute of child health, LONDON, UK

## Introduction

Cryopyrin associated periodic syndromes (CAPS) are caused by autosomal dominant gain of function mutations in the NLRP3 gene. However, up to 50% of clinically diagnosed CAPS patients with typical clinical features and good response to anti-IL-1b treatment have no mutation detected by conventional Sanger DNA sequencing. Recent studies suggest that somatic *NLRP3* mosaicism may account for a proportion of these apparently “mutation-negative” patients. Another possible explanation is that CAPS can be caused by other genetic mutations.

## Objectives

The aim of this study was therefore to assess the relative contributions of NLRP3 somatic mosaicism or alternative genetic cause in a cohort of paediatric and adult patients with a clinical diagnosis of CAPS, but who were NLRP3 mutation negative by Sanger sequencing.

## Methods

To detect somatic mosaicism for *NLRP3* we performed massively parallel sequencing (MPS) of *NLRP3* with high coverage of DNA extracted from peripheral blood. In the patients who were negative for somatic mosaicism using MPS, we then went on to perform Whole Exome Sequencing (WES) on DNA from peripheral blood from select cases using the Illumina TruSeq or Nextera Exome capture and HiSeq sequencing platforms. Exome data was analysed in the Galaxy web-based suit.

## Results

Eight patients including 4 children (n=2 with CINCA; n=2 with Muckle-Wells syndrome [MWS]; and 4 adults with late-onset MWS) were studied. MPS analyses revealed a variable degree of somatic *NLRP3* mosaicism in 6/8 (75%) patients: 4 adults and 2 children. Two patients with MWS carried the previously described pathogenic p.E567K *NLRP3* mutation in 3.1% and 5.6% of alleles respectively; one of the CINCA patients had the pathogenic p.F556L NLRP3 mutation in 14.5% of alleles; the other two unrelated adult patients had a novel p.Y563C *NLRP3* mutation in 7.3% and 9.75% of alleles respectively. The last adult patient had the pathogenic p.A352T mutation, previously described as a pathological germline mutation, at an allele frequency of 16.1%.

WES was performed in the remaining 2 patients (n=1 CINCA; n=1 MWS). WES revealed a novel NOD2 mutation in the CINCA-like patient, which was confirmed by Sanger sequencing and segregated with the disease in family studies, thus confirming the diagnosis of Blau’s syndrome rather than CINCA; in the other patient a definite causal mutation is yet to be found using WES.

## Conclusion

Somatic NLRP3 mosaicism accounted for 75% of the “NLRP3 mutation-negative” cases in this cohort which confirms the importance of genetic somatic mutation in the aetiopathogenesis of CAPS. This included 4 adults with adult-onset disease, making this the first description of low-level somatic *NLRP3* mosaicism as a cause of late-onset MWS in adulthood. WES revealed a novel NOD2 mutation in one child with atypical CINCA, emphasizing that there may be a significant clinical overlap between different auto-inflammatory syndromes; for the remaining case of MWS, WES has yet to reveal the genetic cause, and therefore whole-genome sequencing could be indicated.

## Disclosure of interest

None declared.

